# Hit Identification and Functional Validation of Novel Dual Inhibitors of HDAC8 and Tubulin Identified by Combining Docking and Molecular Dynamics Simulations

**DOI:** 10.3390/antiox13111427

**Published:** 2024-11-20

**Authors:** Antonio Curcio, Roberta Rocca, Federica Chiera, Maria Eugenia Gallo Cantafio, Ilenia Valentino, Ludovica Ganino, Pierpaolo Murfone, Angela De Simone, Giulia Di Napoli, Stefano Alcaro, Nicola Amodio, Anna Artese

**Affiliations:** 1 Department of Health Sciences, University Magna Græcia, 88100 Catanzaro, Italy; antonio.curcio@unicz.it (A.C.); f.chiera@unicz.it (F.C.); alcaro@unicz.it (S.A.); artese@unicz.it (A.A.); 2 Net4Science Srl, University Magna Græcia, 88100 Catanzaro, Italy; 3 Associazione CRISEA-Centro di Ricerca e Servizi Avanzati per L’innovazione Rurale, Località Condoleo di Belcastro, 88100 Catanzaro, Italy; 4 Department of Experimental and Clinical Medicine, University Magna Græcia, 88100 Catanzaro, Italy; mariaeugenia.gallocantafio@unicz.it (M.E.G.C.); ilenia.valentino@studenti.unicz.it (I.V.); ludovica.ganino@studenti.unicz.it (L.G.); pierpaolo.murfone@studenti.unicz.it (P.M.); 5 Department of Drug Science and Technology, University of Turin, via Pietro Giuria 9, 10125 Turin, Italy; angela.desimone@unito.it (A.D.S.); giulia.dinapoli@unito.it (G.D.N.)

**Keywords:** HDAC8, tubulin, virtual screening, multi-targets, epigenetics, docking, molecular dynamics

## Abstract

Chromatin organization, which is under the control of histone deacetylases (HDACs), is frequently deregulated in cancer cells. Amongst HDACs, HDAC8 plays an oncogenic role in different neoplasias by acting on both histone and non-histone substrates. Promising anti-cancer strategies have exploited dual-targeting drugs that inhibit both HDAC8 and tubulin. These drugs have shown the potential to enhance the outcome of anti-cancer treatments by simultaneously targeting multiple pathways critical to disease onset and progression. In this study, a structure-based virtual screening (SBVS) of 96403 natural compounds was performed towards the four Class I HDAC isoforms and tubulin. Using molecular docking and molecular dynamics simulations (MDs), we identified two molecules that could selectively interact with HDAC8 and tubulin. CNP0112925 (arundinin), bearing a polyphenolic structure, was confirmed to inhibit HDAC8 activity and tubulin organization, affecting breast cancer cell viability and triggering mitochondrial superoxide production and apoptosis.

## 1. Introduction

Chromatin structure drives the organization of genetic information within a cell, influencing the activation or silencing of gene expression [1]. Epigenetic modifications, including DNA cytosine base methylation, histone post-translational modifications, and nucleosome positioning, play a role in determining which genes are switched on or off and contribute to the inheritance of gene expression patterns. The improper maintenance of these marks can lead to the dysregulation of signaling pathways, contributing to diseases such as cancer [2,3].

Among histone modifications, acetylation plays a crucial role in regulating gene expression, generally leading to increased transcription, while deacetylation is often associated with gene repression.

Histone deacetylases, commonly referred to as HDACs, are enzymes that play a crucial role in regulating genes by eliminating acetyl groups from histones [4]. This process leads to chromatin condensation, which significantly alters gene transcription.

HDACs also play a significant role in the development of drug resistance mechanisms in tumor cells [5,6,7]. There are 4 classes and 18 different isoforms within the HDAC family. Classes I, II, and IV are zinc-dependent metalloproteins, while Class III relies on NAD^+^ as a cofactor. Class I, located mainly in the nucleus, includes the isoforms 1, 2, 3, and 8. They are characterized by a smaller size (350–500 amino acids) and are primarily implicated in cancer progression [8]. Regarding the Class I HDAC family, HDACs 1–3 form at least five extensive multi-protein co-repressor complexes. Chromatin recruits these complexes through interactions with repressive transcription factors or other silencing co-factors [9]. Conversely, HDAC8 is the only Class I HDAC that remains fully active in isolation and does not join a larger complex [10].

The overexpression of HDACs has been observed in different types of cancer compared to corresponding tissues and is related to a poor prognosis [11]. Elevated levels of HDACs in tumor cells lead to aberrant histone deacetylation, resulting in the silencing of genes essential for correct cell differentiation and growth; in addition, HDAC inhibitors can mediate the induction of a variety of cell death mechanisms, which correlate to the anti-cancer activity in several cancer cell models [12]. In 2006, Vorinostat (SAHA, Suberoylanilide Hydroxamic Acid) became the first FDA-approved HDAC inhibitor for cutaneous T-cell lymphoma [13]. However, early studies suggest that pan-HDAC inhibitors may cause various side effects, including bone marrow depression, diarrhea, weight loss, taste disturbances, electrolyte changes, disordered clotting, fatigue, and cardiac arrhythmias [14]. Given the critical role of HDACs in chromatin structure and protein modification, the future development of drugs targeting specific HDAC isoforms with predominant oncogenic functions in tumor cells is increasingly necessary [15,16].

Since its discovery in 2000 as the latest member of Class I HDACs, HDAC8 has undergone extensive structural and functional studies [17,18], which shows that it plays a pivotal role in human pathophysiology [19,20]. In cancer, HDAC8 has been shown to promote growth, metastasis, and immune evasion; consistently, its genetic ablation or pharmacological inhibition elicited anti-cancer effects across various cancer types [21,22,23,24,25]. Structurally, HDAC8 forms a head-to-head dimer consisting of two nearly identical molecules. Each molecule contains one zinc-binding site, promoting catalytic activity, and two potassium-binding sites, which enhance structural stability [22]. The active site of HDAC8 includes a hydrophobic tunnel [19] made up of six residues (PHE 152, PHE 208, TYR 306, GLY 151, HIS 180, and MET 274), interacting with four methylene groups of the substrate. At the end of the tunnel, a catalytic machinery that coordinates the deacetylation process is located. In particular, the zinc ion, bound to ASP 178, ASP 267, and HIS 180, coordinates the deacetylation process with the residue TYR 306 by activating the substrate amide carbonyl (C=O) for a nucleophilic attack. ASP 101 is essential for substrate binding by directing conformational changes in the L2 loop from an unbounded to a bound state [26].

Recently, multi-target therapy has emerged as an effective strategy to achieve higher therapeutic efficacy in various disease settings, in particular exploiting dual-target drugs [27]. Combining HDAC inhibitors with other anti-tumor agents, including microtubule-targeting agents (MTAs), appears to be a rational strategy to improve the effectiveness of single-target drugs for cancer treatment due to their synergistic effect on cellular processes in cancer cells [28].

Microtubules are widespread structural components of the cytoskeleton, assembled through the self-organization of tubulin heterodimers. The α and β tubulin proteins, each consisting of approximately 450 amino acids, exhibit high homology. Each monomer in the α/β-tubulin heterodimer binds to a guanine nucleotide (GTP), crucial for microtubule assembly. Indeed, the hydrolysis of GTP occurs only after the addition of the α/β-tubulin dimer to the growing microtubule structure [29]. Various natural and synthetic compounds, including taxanes, vinca alkaloids, macrolides, and peptides, are recognized for their ability to disrupt microtubule dynamics. These ligands, after binding to tubulin, interfere with microtubule dynamics within cells, inhibiting cell division at mitosis and resulting in cell death. Consequently, MTAs have been widely employed as experimental tools to investigate the role of microtubule dynamics in various cellular processes [30,31,32,33,34].

On the basis of the above-reported findings, we performed a structure-based virtual screening (SBVS) of natural compounds acting as dual binders for HDAC8 and tubulin by evaluating their theoretical affinity for the zinc-dependent catalytic site of all Class I HDACs and the colchicine site, located at the intrasubunit interface within the α/β tubulin dimer. One of these natural compounds, arundinin, found in *Pleione bulbocodioides*, *Pleione yunnanensis*, and other organisms, was demonstrated to significantly inhibit HDAC8 activity and to affect tubulin organization, with effects on mitochondrial superoxide and apoptosis on breast cancer cells.

## 2. Materials and Methods

### 2.1. Proteins Preparation and Docking Simulations

In our SBVS study, we carefully selected and downloaded the 3D coordinates of tubulin and of each Class I HDAC isoform from the Protein Data Bank (PDB) [35]. In particular, we used the crystallographic structures with the following PDB codes: 4BKX for HDAC1 [36]; 4LXZ for HDAC2 [37]; 4A69 for HDAC3 [38]; 1T64 for HDAC8 [19]; and 4O2B for tubulin [30]. We selected protein structures based on three factors: (1) high resolution, (2) minimal missing regions, and (3) the absence of mutations and covalently bound ligands. For HDAC2, HDAC8, and tubulin, we favored models that included co-crystallized inhibitors, as they provide valuable information about ligand binding and protein function. Conversely, for HDAC3, we had to rely on the single available structure in the PDB due to the lack of alternative options.

The structures were prepared and energy-optimized through the Protein Preparation Wizard v4.1 [39] tool implemented in Maestro v4.1 [40], using OPLS_2005 as a force field [41]. In particular, residual crystallographic buffer components and water molecules were removed, hydrogen atoms were added, and side chains’ protonation states were assigned at pH 7.4 [39].

Since HDAC isoforms 1 and 3 do not have a co-crystallized ligand, their rigid receptor grid was constructed by centering the 10 × 10 × 10 Å inner box on the three residues of the active site most involved in the deacetylation process. Specifically, for HDAC1, the key residues are HIS 140, HIS 141, and TYR 303, while for HDAC3, the corresponding residues are HIS 134, HIS 135, and TYR 298. Albeit the co-crystalized ligands were complexed to HDAC2 and HDAC8 isoforms, in order to apply the same procedure, we centered the inner grid box on HIS 141, HIS 142, and TYR 304 for HDAC2 and on HIS 142, HIS 143, and TYR 306 for HDAC8, respectively. Therefore, the Glide SP protocol v7.8 was applied while maintaining the default parameters to produce ten ligand poses [42]. We performed a preliminary validation of the computational docking protocol using the grids of HDAC2 and HDAC8, aiming to evaluate its ability to accurately reproduce the crystallographic pose of the reference compounds (Table S1).

Then, we created a receptor grid for each of the two α/β dimers of the tubulin model by aligning the inner box with the co-crystallized colchicine. After the redocking procedure, we obtained the best RMSD value for the A/B dimer, which was selected for further screening investigation (Table S2). To identify the most promising *hits*, we used the D-Score values. For each HDAC, we employed the best D-Score of Trichostatin A or Vorinostat as cut-off values (Table S3). Similarly, we used the D-Score value of the best-redocked pose of colchicine (−10.548 kcal/mol) as the cut-off for tubulin. These values helped us to select the most interesting *hits* during our screening process.

### 2.2. Database Preparation

In this SBVS study, we screened the COCONUT database [43], including 407,270 natural compounds. We selected 96,403 compounds without centers of asymmetry, and we prepared them through the LigPrep Tool v4.5 [44]. Thus, hydrogens were added, salts were removed, and ionization states were calculated using Epik at pH 7.4. Each structure was submitted to the default energy minimization steps of LigPrep using OPLS_2005 as a force field [41]. After the SBVS, we selected only those compounds whose D-Score respected the *cut-off* for HDAC8 and tubulin. This procedure was performed with the aim to identify selective dual HDAC8/tubulin binders. Afterwards, we applied a further pharmacokinetic filter using the QikProp tool v5.5 [45] and we kept only the compounds able to comply with the five Lipinski rules. The correct protonation state of these compounds was thoroughly investigated using the MSketch tool v16.3.28 [46], and subsequently, a fingerprint clustering of these compounds was performed adopting the Tanimoto coefficient. The number of clusters was quantified using the Kelly criterion, obtaining 4 clusters [47]. Finally, for each cluster, the molecule with the greatest number of shared features was selected based on the centroid.

### 2.3. Molecular Dynamics Simulations (MDs)

The complexes of the 4 selected molecules with all the HDAC isoforms and the tubulin were submitted to 200 ns of MD simulations using Desmond ver. 4.4 [48], with OPLS_2005 as a force field [41]. As a positive control, we also submitted to MDs the pan-HDAC inhibitors (Vorinostat and Trichostatin A) and the colchicine complexed to all the Class I HDAC isoforms and the tubulin, respectively. All systems were placed in a 10 Å layer orthorhombic box in explicit solvent with TIP3P [49] water model parameters. For each system, counterions were added until charge neutralization. The RESPA integrator was applied with different time steps for various interactions: 2 fs for bonded and near-range interactions and 6 fs for non-bonded (far) interactions. For short-range Coulombic interactions, a time step of 1 fs was used, along with a 9.0 Å cut-off distance. In contrast, long-range Coulombic interactions were addressed using the Smooth Particle Mesh Ewald (PME) method [50]. After optimizing the solvated model, the systems were relaxed with the Martyna–Tobias–Klein isobaric–isothermal ensemble (MTK_NPT) and then equilibrated through the NVT ensemble at 10 K by using the NPT ensemble at 300 K and 1 atm with the Berendsen thermostat–barostat. Trajectory frames were collected every 200 ps and analyzed by means of the Simulation Interaction Diagram (SID) in order to geometrically and thermodynamically investigate the obtained trajectories. To confirm the selectivity for isoform 8, we analyzed the ability of the compounds to establish coordination bonds with the zinc ion.

### 2.4. HDACs Inhibition In Vitro Assay

HDAC8 isoform was purchased from VinciBiochem (VinciBiochem, Florence, Italy). White 96-well plates were purchased from Millipore (Millipore Iberica S.A.U.). HDAC-Glo (TM) I/II Assay kit was obtained from Promega (Promega, Madison, WI, USA).

A bioluminogenic assay was used to monitor the activity of the HDAC8 enzyme. A proluminogenic substrate containing an acetylated lysine peptide sequence derived from histone 4 conjugated to aminoluciferin was applied. HDAC enzyme–mediated deacetylation of the lysine residue facilitates luminogenic substrate susceptibility to specific proteolytic cleavage by the enzyme in the developer reagent [51]. The aminoluciferin product obtained from the cleavage is a luciferase substrate, and the amount of light produced in this reaction is proportional to enzyme activities. The HDAC-Glo I/II assay reagent was prepared by (i) rehydration of lyophilized HDAC-Glo I/II substrate (with an acetylated peptide concentration of 100 μM) in 10 mL HDAC-Glo I/II assay buffer and (ii) addition of 10 μL of developer reagent (containing trypsin). The % of inhibition as well as the IC_50_ values for both standard inhibitor Trichostatin A and CNP0112925 (arundinin) and CNP0217284 compounds towards HDAC8 were determined by diluting HDAC enzymes as appropriate, using the HDAC-Glo I/II assay buffer. 25 μL of solution containing enzyme (1000 ng/mL) was dispensed into microtiter plates. Then, the same volume of HDAC-Glo I/II assay buffer in the absence of tested compounds (activity) and in the presence of inhibitors at desired concentrations was added. After a 30 min incubation time at 37 °C, the enzymatic reaction was stopped by adding 50 μL of developer reagent prepared as reported previously. The microtiter plate was mixed briefly by orbital shaking (500–700 rpm), and luminescence was measured after 15 min using a Victorx5 (PerkinElmer Waltham, MA, USA) plate reader. For inhibitors concentration-response experiments, the IC_50_ values were calculated by fitting the duplicate data in GraphPad Prism (version 10.0; GraphPad Software, La Jolla, CA, USA).

### 2.5. Cell Line and Culture Conditions

MCF7 and MDA-MB-453 cell lines were purchased from ATCC (Manassas, VA, USA) and cultured in Dulbecco’s modified eagle medium (Corning, New York, NY, USA) supplemented with 10% heat-inactivated fetal bovine serum (FBS) (Gibco–Thermo Fisher Scientific, Waltham, MA, USA), penicillin (100 I.U/mL), and streptomycin (100 ng/mL) (Sigma, Burlington, MA, USA). The cells were cultured at 37 °C in a humidified incubator with 5% CO_2_.

### 2.6. Cell Viability Assay

Cell viability was assessed using the Cell Titer Glo (CTG) assay (Promega, Madison, WI, USA), according to the manufacturer’s instructions. Briefly, MCF-7 and MDA-MB-453 cell lines were plated in a 96-well plate and treated with different concentrations of arundinin. At established time points, the single reagent provided by the kit was added to the cells for simultaneous cell lysis and generation of a luminescent signal proportional to the ATP content, which correlates with the number of viable cells in the culture. Luminescence was measured by using the GloMax multi-detection system (Promega, Madison, WI, USA). 

Half-maximal inhibitory concentration (IC_50_) of arundinin was determined, in three independent experiments, for both MCF7 and MDA-MB-453 cell lines using GraphPad Prism.

### 2.7. Measurement of Mitochondrial ROS

Intracellular mitochondrial ROS levels were assessed using the Mito-SOX Red mitochondrial superoxide indicator (Invitrogen, Waltham, MA, USA). MCF-7 cells were harvested after 48 h of arundinin treatment then washed in PBS and stained with 1 µM Mito-SOX Red in PBS for 30 min at 37 °C in the dark. After incubation, the cells were washed and analyzed using flow cytometry. All experiments were performed in triplicate and acquired by using FACS Fortessa X-20 (BD Biosciences, Franklin Lakes, NJ, USA). For each sample, at least 1 × 10^4^ events were acquired.

### 2.8. Determination of Mitochondrial Membrane Potential

The mitochondrial membrane potential was evaluated using the fluorescent dye Tetramethylrhodamine, Methyl Ester, and Perchlorate (TMRM). After treatment with arundinin, MCF7 and MDA-MB-453 BC cell lines were incubated with a staining solution containing 100 nM TMRM (Thermo Fisher Scientific, Waltham, MA, USA) in PBS for 30 min at 37 °C to allow dye diffusion across the plasma membrane and the inner mitochondrial membrane. Following incubation, cells were washed and analyzed by flow cytometry. All experiments were performed in triplicate and acquired by using FACS Fortessa X-20 (BD Biosciences, Franklin Lakes, NJ, USA). For each sample, at least 1 × 10^4^ events were acquired.

### 2.9. Detection of Apoptosis

Apoptosis was investigated by using Annexin V/7-AAD flow cytometry assay, as reported [52]. MCF-7 cells were seeded in a 24-well plate and treated with different doses of arundinin. Cells were harvested in 5 mL polystyrene tubes and stained by using a PE-Annexin V Apoptosis Detection kit (Invitrogen, Waltham, MA, USA), according to the manufacturer’s protocol. In detail, MCF-7 cells were washed twice in cold PBS 1×, resuspended in Binding Buffer 1x, and stained with PE-Annexin V and 7-AAD probes. After 15 min of incubation in the dark at room temperature, cells were analyzed by using FACS Fortessa X-20 (BD Biosciences). For each sample, at least 1 × 10^4^ events were acquired.

### 2.10. Western Blot Analysis (WB)

Protein extraction and Western blot analysis were performed according to standard protocol, as reported [53]. Briefly, MCF-7 cells were lysed in NP-40 lysis buffer (Thermo Fisher Scientific), containing Halt Protease and Phosphatase Inhibitor Single-Use cocktail (100×, Thermo Fisher Scientific). The whole cell lysate was separated using 10% SDS-Acrylamide gels (Bio-Rad, Hercules, CA, USA) and electro-transferred on Nitrocellulose membranes (Bio-Rad, Hercules, CA, USA). The membranes were blocked for 1 h in 5% milk and immunoblotted overnight at 4 °C with each of the following primary antibodies: PARP1 (#9542) and Caspase 3 (#9655S), both from Cell Signaling Technology (Danvers, MA, USA); α-Tubulin antibody (#2125S), used as loading control, was from Cell Signaling Technology; BAX was SantaCruz Biotechnology (Dallas, TX, USA) (sc-20067); BCL2 (MA5-11757) was from Invitrogen, Thermofisher Scientific (Waltham, MA, USA).

Chemiluminescence was detected using the Clarity Western ECL Substrate (Biorad Laboratories, Hercules, CA, USA).

### 2.11. Immunofluorescence (IF) Microscopy

MCF7 cells were grown on poly-L-lysine-coated coverslips and treated with 25 µM arundinin for 48 h. After incubation, cells were fixed in 4% paraformaldehyde for 20 min at room temperature, permeabilized with 0.2% Triton X-100 in PBS for 15 min then blocked for 1 h with 1.5% BSA in PBS and incubated overnight at 4 °C with α-Tubulin primary antibody (#sc-5286). Subsequently, MCF-7 cells were washed in PBS and incubated for 1 h with fluorescent goat anti-mouse secondary antibody (Alexa Fluor 488; Invitrogen, 1:1000). After washing, coverslips were mounted on a glass slide with DAPI-containing mounting medium (Santa Cruz Biotech., Dallas, TX, USA), for nuclear staining. Images were obtained under the Leica DM4 B fluorescence microscope.

## 3. Results

### 3.1. Molecular Modeling Analysis

We prepared the models of the four distinct HDAC isoforms and tubulin, and we downloaded the COCONUT database [43], a comprehensive resource of natural compounds, including 407,270 molecules. Next, we selected and screened the 96,403 compounds without centers of asymmetry, avoiding the complexities of stereochemical control and concerns over enantiomeric purity. To establish the cut-off value for the SBVS, we first calculated the D-Score value for each HDAC isoform using the best-docked pose for two pan HDAC inhibitors, Vorinostat and Trichostatin A (Table S3). For all HDAC isoforms, Trichostatin A reached the best D-Score value thus we used this value as the cut-off point for the subsequent SBVS. Additionally, we selected the D-Score value of the best re-docked pose of colchicine (Table S2).

**Figure 1:**
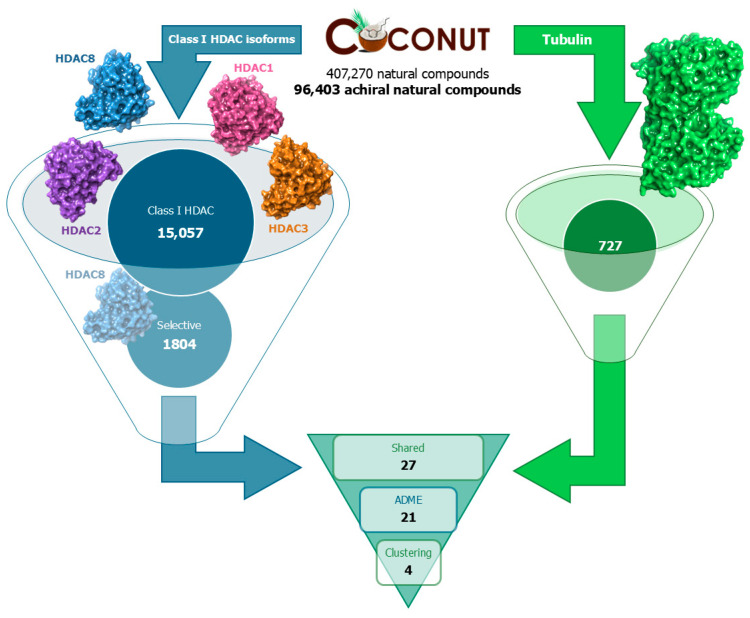
Structure-based virtual screening (SBVS) workflow.

After SBVS, it emerged that 15,057 compounds fell within the cut-off value on at least one of the HDAC analyzed isoforms, and of these, 5042 had a D-Score value better than the cut-off for the isoform of our interest. From the docking simulations, the number of compounds potentially selective for HDAC8, respecting the cut-off only on this isoform, decreased to 1804. Regarding the tubulin target, 727 compounds fitted the cut-off. Out of these, 27 molecules showed a D-Score value lower than the cut-off only for HDAC8, indicating a potential to selectively bind to both HDAC8 and tubulin. After applying an additional pharmacokinetic filter using the QikProp tool, we retained only compounds that complied with the five Lipinski rules. We then investigated their correct protonation state using the MSketch tool and reduced the number of molecules to 21 (Figure 1).

**Figure 2:**
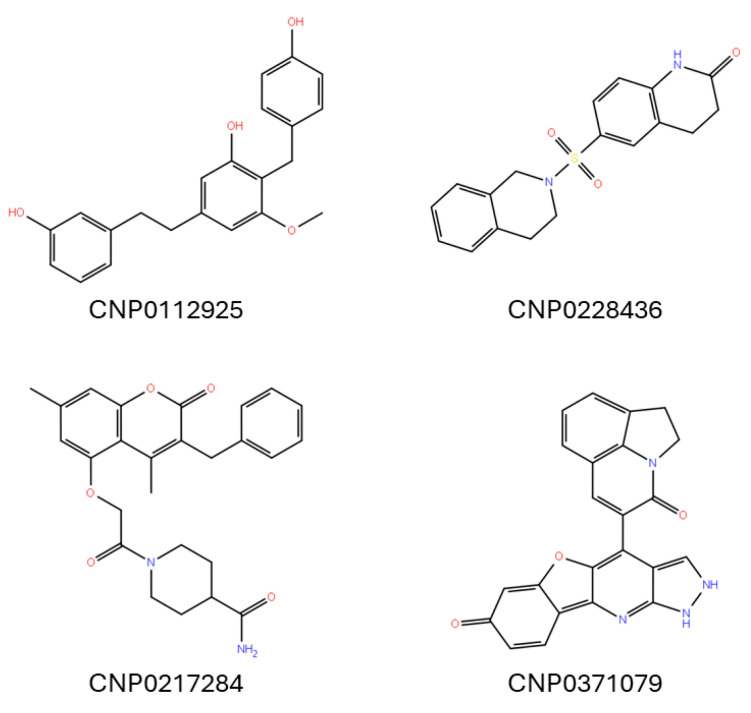
Two-dimensional structures of the final *hits* proposed as potential dual binders of HDAC8/tubulin.

The selected compounds underwent fingerprint clustering utilizing the Tanimoto coefficient. The number of clusters was determined using the Canvas tool v3.9 [47], guided by the Kelly criterion, which resulted in four distinct clusters. In the final step of the selection process, we identified the molecule with the largest number of features within each cluster. This was accomplished by evaluating the centroid of each molecule and comparing it to the features of other molecules in that cluster. Once the molecule with the highest number of features was identified, it was selected for further analysis. The results of this process are reported in Figure 2 and Table 1.

The analysis of the four final *hits* highlighted that they all exhibited good D-Score values when interacting with either HDAC8 or tubulin. Specifically, these *hits* showed D-Scores ranging from −8.05 to −8.48 kcal/mol when interacting within the HDAC8 binding pocket and from −10.64 to −10.79 kcal/mol when interacting with tubulin (Table 1). Conversely, for the other HDAC isoforms, the obtained D-Score values were less promising compared to HDAC8 and tubulin.

Regarding the four selected natural compounds, their binding mode on HDAC8 and tubulin was analyzed in detail.

**Figure 3:**
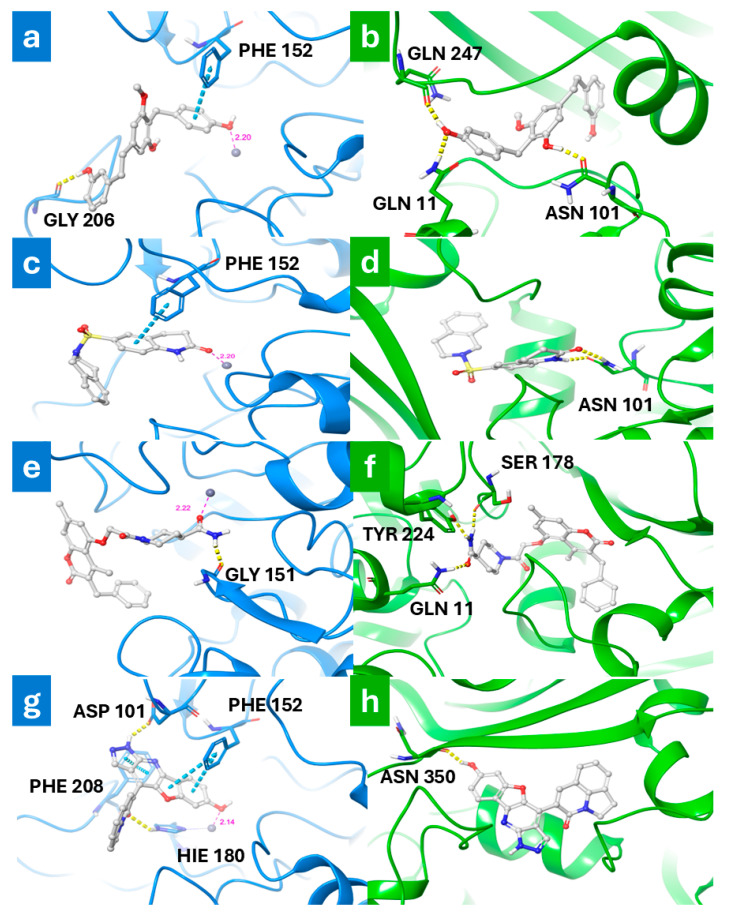
Three-dimensional structure of the best binding pose generated by molecular docking simulation for the 4 *hits* with HDAC8 (light blue) and tubulin (green): (**a**,**b**) CNP0112925, (**c**,**d**) CNP0228436, (**e**,**f**) CNP0217284, and (**g**,**h**) CNP0371079.

The compound with COCONUT ID CNP0112925, known as arundinin, is a natural product found in *Pleione bulbocodioides*, *Pleione yunnanensis,* and other organisms. This compound has a polyphenol structure and has been shown to possess anti-inflammatory properties in human neutrophils [54]. In the best binding pose within the HDAC8 deacetylation site, the ligand is involved in a hydrogen bond (H-bond) with GLY 206, which anchors it into the site, and in π-π stacking with PHE 152 (Figure 3a). Additionally, the phenolic group of the ligand was found in close proximity to the zinc ion at a distance of only 2.20 Å, thus establishing effective electrostatic interactions. On the other hand, in the colchicine binding site of the tubulin (Figure 3b), the compound is involved in three H-bonds with the side chain of GLN 11 and ASN 101 and with the backbone of GLN 247.

The compound identified by the COCONUT ID CNP0228436 has a dihydro-isoquinolin-2-ylsulfonyl-dihydro-quinolin-2-one structure that is essential for interacting with both targets. Specifically, the docking results revealed that in the active site of HDAC8, it establishes a π-π stacking interaction with PHE 152 (as shown in Figure 3c). In the colchicine binding site, it forms two H-bonds with ASN 101 (as reported in Figure 3b). Moreover, its carbonyl group is positioned 2.20 Å away from the divalent cation of HDAC8, enabling the formation of electrostatic interactions.

The docking analysis of the compound identified by COCONUT ID CNP0217284 revealed that the 2-oxo-2H-chromene scaffold prevents entry into the active site of HDAC8 (Figure 3e). In contrast, the piperidine-4-carboxamide group is located within the active site and interacts through electrostatic interactions with the zinc ion at a distance of 2.22 Å. Furthermore, the amide group engages an H-bond with the backbone of GLY 151 (Figure 3e). In the colchicine site of tubulin (Figure 3f), the carboxamide group of the compound is involved in three H-bonds with the side chains of GLN 11 and TYR 224, as well as with the backbone of SER 178. Additionally, the coumarin unit establishes numerous hydrophobic contacts.

Finally, the compound with COCONUT ID CNP0371079 displays a large polycyclic structure that creates multiple favorable interactions in the deacetylation site, as shown in the best docking pose with HDAC8 (Figure 3g). Specifically, there are two π-π interactions between the 7-hydroxy-1H-benzofuric rings and the side chain of PHE 152, as well as another π-π interaction between the pyridine and the side chain of PHE 208. Additionally, two H-bonds are formed between the pyrazolic ring and the quinolone portion of the ligand with the side chain of ASP 101 and HIS 180, respectively. The hydroxyl group is positioned 2.14 Å away from the zinc ion, allowing favorable electrostatic interactions. In contrast, in the colchicine binding site of tubulin (Figure 3h), the molecule’s hydroxyl group establishes only one H-bond with the backbone of ASN 350. Nevertheless, several hydrophobic contacts are able to stabilize the complex.

To explore the molecular recognition process against HDAC8 and tubulin and to assess selectivity towards the relevant HDAC isoform, the complexes of the four selected compounds were submitted to explicit water solvent molecular dynamics simulations (MDs). In order to verify the reliability and validity of the protocol, MDs were conducted on Vorinostat and Trichostatin A, two pan-HDAC inhibitors, against the four Class I HDAC isoforms. Additionally, MDs were also performed on colchicine against tubulin. These investigations were carried out to ensure that the protocol can accurately and effectively predict the behavior of these compounds. MDs confirmed that Vorinostat and Trichostatin A bind to all the analyzed HDAC isoforms in a non-selective manner. During MDs, the stability of the ligand binding mode was evaluated by analyzing the Root-Mean-Square Deviation (RMSD) calculated on the heavy atoms of the ligand (all atoms except hydrogens), initially aligning the complex on the protein backbone of the first MD frame structure. Both pan-HDAC inhibitors exhibited a low RMSD in all four HDAC analyzed isoforms, indicating high geometrical stability of their binding mode. Furthermore, they were observed to interact and coordinate zinc throughout the simulation on each studied isoform (Figures S1 and S2).

**Figure 4:**
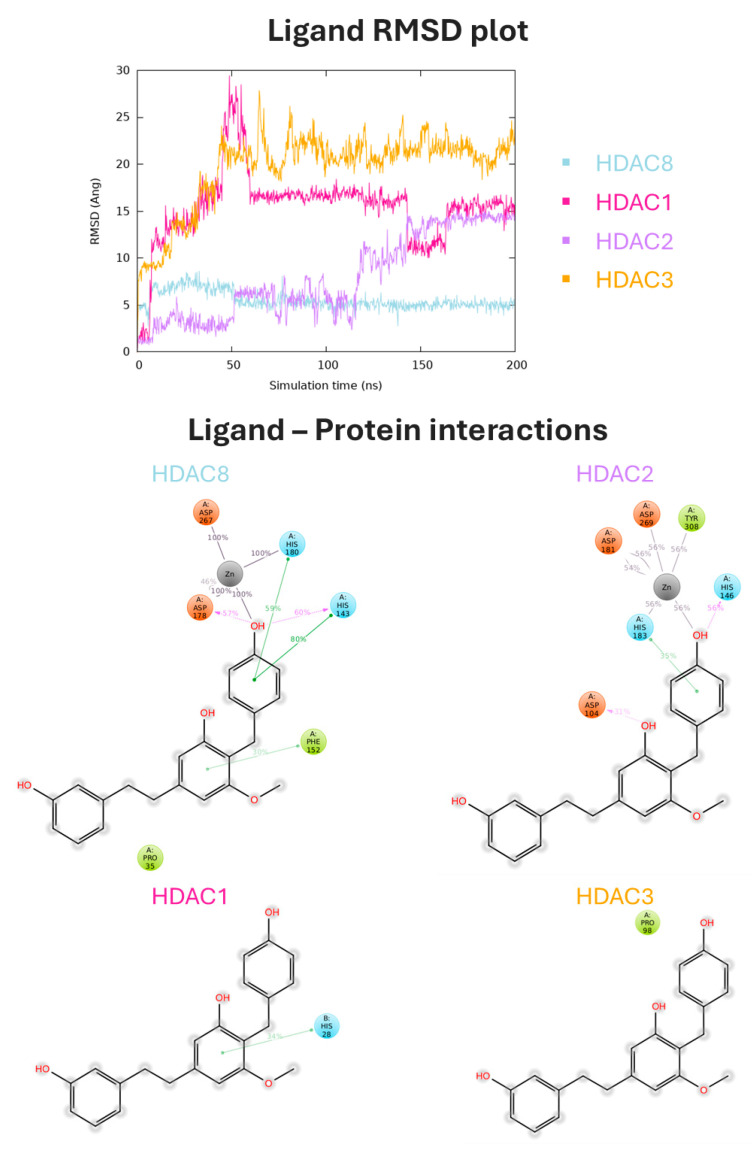
MD analysis for arundinin (CNP0112925) in complex with Class I HDACs. In the top panel, the ligand RMSD plot, expressed in Å and calculated on the heavy atoms of the ligand, is reported. In the bottom panel, ligand atom interactions with the protein residues of HDAC8 (light blue), HDAC1 (pink), HDAC2 (purple), and HDAC3 (orange) are indicated. Only interactions that occur more than 30.0% of the simulation time in 200 ns of the trajectory are shown.

The results of MDs on the four selected natural compounds were examined in detail. In particular, our analysis revealed that two of the selected compounds (CNP0228436 and CNP0371079) exhibited a potentially low selectivity towards HDAC8. This was indicated by the low RMSD values observed for these compounds when bound to HDAC1 and HDAC2 (Figures S3 and S4). On the other hand, arundinin (CNP0112925) and CNP0217284 exhibited a good stability and selectivity profile towards HDAC8. Both compounds were associated with low RMSD values on HDAC8 compared to other isoforms, maintaining the electrostatic interaction with the zinc ion exclusively in the HDAC8 complex. Arundinin showed high RMSD values in HDAC1 and HDAC3 complexes, quickly leaving the binding site, as shown by the analysis of the ligand interactions. When complexed with HDAC2, the compound changed its pose in the active site and lacked interaction with the divalent cation. Lastly, when complexed with HDAC8, the compound was stable during the entire simulation, maintaining stable contact with the phenolic group and the zinc ion. Its phenolic group also engaged two H-bonds with ASP 178 and HIS 143. The complex was further stabilized by three π-π interactions with HIS 143, PHE 152, and HIS 180. These interactions were found to have a percentage of 80%, 30%, and 59%, respectively (Figure 4).

**Figure 5:**
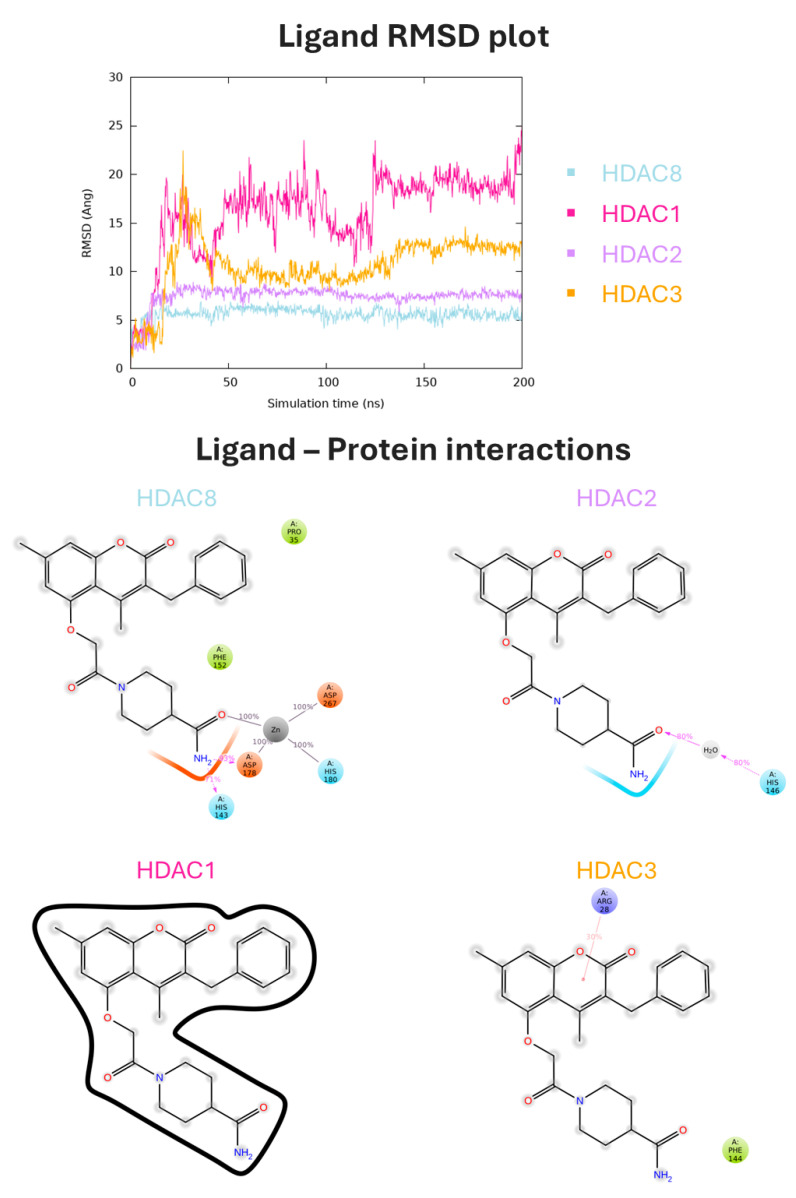
MDs analysis for compound CNP0217284 in complex with Class I HDACs. In the top panel, the ligand RMSD plot, expressed in Å and calculated on the heavy atoms of the ligand, is reported. In the bottom panel, ligand atom interactions with the protein residues of HDAC8 (light blue), HDAC1 (pink), HDAC2 (purple), and HDAC3 (orange) are indicated. Only interactions that occur more than 30.0% of the simulation time in 200 ns of the trajectory are shown.

Finally, compound CNP0217284 was associated with a low RMSD on HDAC8 compared to other isoforms and maintained the electrostatic interaction with the zinc ion exclusively in the HDAC8 complex. During the MDs, the amide portion of the compound was involved in an H-bond with HIS 143 for 71% of the time. Additionally, it exhibited hydrophobic interactions with PRO 35 and PHE 152 (Figure 5).

**Figure 6:**
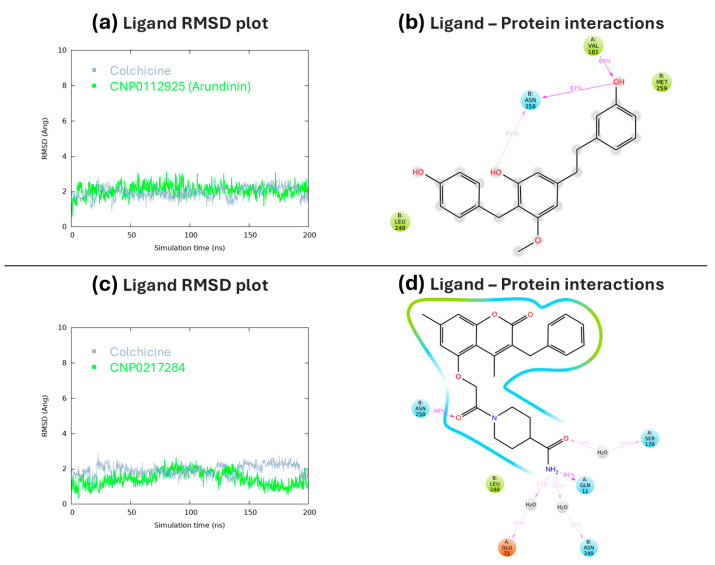
RMSD plots of colchicine and CNP0112925 (**a**), and colchicine and CNP0217284 (**c**) in complex with tubulin. RMSD values are expressed in Å and calculated on the heavy atoms of the ligand. Two-dimensional representation of ligand atom interactions of CNP0112925 (**b**) and CNP0217284 (**d**) with the protein residues of tubulin. Only interactions that occur more than 30.0% of the simulation time in 200 ns of the trajectory are shown.

Thus, CNP0112925 and CNP0217284 potentially resulted as the most selective for HDAC8 were submitted to MDs in a complex with tubulin. Interestingly, both compounds showed remarkable stability when bound to tubulin, indicating their potential use as dual inhibitors of HDAC8/tubulin. Both compounds exhibited RMSD values comparable to that of colchicine, with arundinin demonstrating notably high stability (Figure 6a–c). During the MDs, arundinin interacted with ASN 258 and VAL 181 by forming two and one H-bonds, respectively (Figure 6b). Meanwhile, compounds CNP0112925 (arundinin) and CNP0217284 formed an H-bond with GLN 11 and ASN 258, respectively. The complex between compound CNP0217284 and tubulin was further stabilized by three water bridges with GLU 71, SER 178, and ASN 249 (Figure 6d).

### 3.2. Functional Validation of the Best Hits

The ability of both CNP0112925 (arundinin) and CNP0217284 to inhibit HDAC8 was investigated by carrying out a luminometric assay. The obtained results, reported in Table 2, indicate that CNP0112925 (arundinin) was able to inhibit HDAC8 enzymatic activity at an IC_50_ approximately 8-fold lower than CNP0217284.

**Figure 7:**
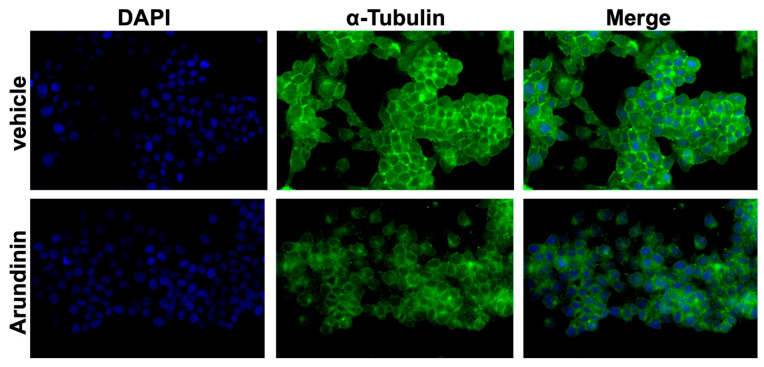
Fluorescence microscopy analysis of MCF7 cells stained for α-tubulin (AF-488) 48 h after 25 µM arundinin treatment; cell nuclei are evidenced by 4′,6-diamidino-2-phenylindole dihydrochloride (DAPI). The scale bar is 100 µm. Representative images are reported.

Subsequently, the effect of arundinin on the microtubule cytoskeleton organization was assessed by an immunofluorescence assay using MCF7 breast cancer (BC) cells as a model. As shown in Figure 7, when MCF7 cells were treated with arundinin, the densities of fibrous microtubule structures (green) were significantly reduced; in contrast, the microtubule cytoskeleton exhibited normal organization after treatment with vehicle (DMSO). These results indicated that arundinin could penetrate into MCF7 cells and inhibit tubulin polymerization.

Based on the obtained experimental assays, the stability of arundinin in the HDAC8 catalytic site and its interaction with the zinc cation, as well as the colchicine binding site of the tubulin, was further assessed by means of triplicate MDs. As reported in Figures S5 and S6, in all the analyzed complexes arundinin maintained a great stability and preserved its interactions with the HDAC8 cation throughout the whole simulations.

### 3.3. In Vitro Anti-Tumor Effects of CNP0112925 (Arundinin) on Breast Cancer Cells

Next, we investigated the in vitro anti-tumor activity of CNP0112925 (arundinin) against two BC cell lines, namely MCF7 and MDA-MB-453.

**Figure 8:**
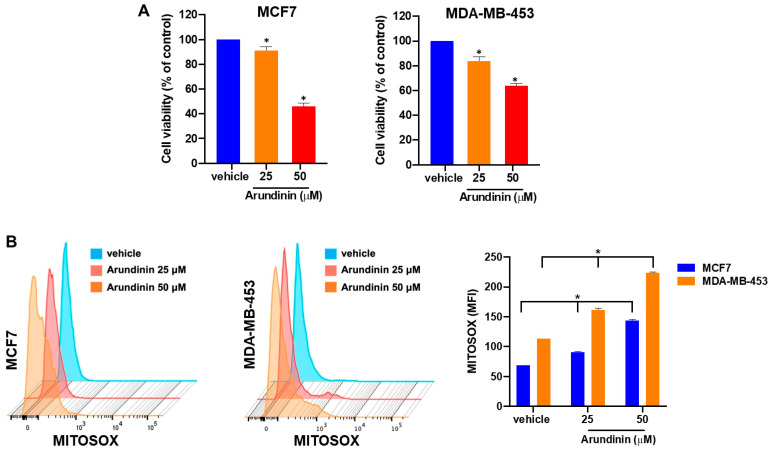
(**A**) Cell viability assessed by Cell Titer Glo assay, 48 h after treatment with 25 µM or 50 µM arundinin; viable cells are represented as a percentage of vehicle-treated cells. * *p*  <  0.05. (**B**) FACS analysis of mitochondrial superoxides measured by Mitosox Red staining in MCF7 or MDA-MB-453 cells treated with DMSO or arundinin for 48 h. Representative fluorescence histograms are reported; results from three independent experiments are reported in the graph. * *p*  <  0.05.

Of note, arundinin treatment led to decreased viability of both BC cell lines (Figure 8A; Supplementary Table S4), triggering mitochondrial dysfunction, as demonstrated by the increase in the production of mitochondrial superoxide anions (MITOSOX) (Figure 8B).

**Figure 9:**
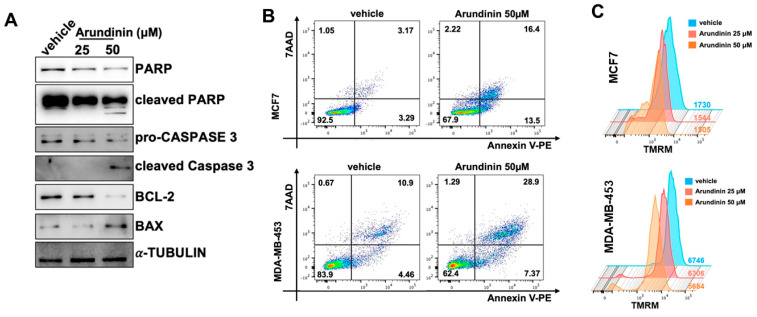
(**A**) WB of full-length and cleaved PARP1, full length and cleaved Caspase-3, Bcl2, and Bax proteins in MCF7 cells, 48 h after 50 µM arundinin treatment; tubulin was used as a loading control. (**B**) Dot plots showing flow cytometric analysis of Annexin V/7-AAD positive BC cells, 48 h after 50 µM arundinin treatment. (**C**) FACS analysis of mitochondrial membrane potential measured by TMRM staining; the data shown are from an independent biological replicate (n = 3).

Inhibition of cell viability could be ascribed to the induction of mitochondrial apoptosis, as demonstrated by the increase in BAX and active caspase 3 and PARP, along with the downregulation of anti-apoptotic BCL2 in WB assays (Figure 9A).

FACS analysis confirmed the occurrence of early and late apoptosis, as evidenced by the increase in Annexin V-positive and Annexin-V/7AAD-positive cells 48 h after treatment of MCF7 and MDA-MB-453 cells with arundinin (Figure 9B). In line with the collapse of mitochondrial membrane potential occurring during apoptosis, BC cells treated with Arundinin were less positive to TMRM dye staining (Figure 9C).

Altogether, these results indicate that arundinin anti-tumor effects are triggered by harmful mitochondrial ROS production and apoptosis.

## 4. Discussion

Recent studies have shown that multi-target therapy is an effective strategy to achieve higher therapeutic efficacy, more specifically using dual-target drugs [55]. In recent years, there has been a growing interest among researchers in natural compounds as a source of bioactive agents with therapeutic potential against various diseases, including cancer.

Overall, natural products have shown to play a relevant role in cancer therapy, with an increasing number of preclinically or clinically used anti-cancer agents being either natural or derived from natural products and capable of impacting diverse pathways in tumor cells [56,57,58,59,60,61,62,63,64,65].

Recent investigation has focused on dual-target binders, which have the ability to simultaneously bind to two different targets, thereby enhancing their therapeutic efficacy. This approach has gained increasing attention due to its potential to overcome the limitations of single-target therapeutics, thus providing new avenues for the development of more effective treatments. As such, the identification and characterization of natural compounds with dual-target binding activity has become an important area of research in the field of drug discovery [66].

In this context, dual binder small molecules that selectively interact with HDAC8 and tubulin, both involved in the apoptotic process, can be considered an innovative strategy for cancer therapies [67]. The application of computational methods, such as Structure-Based Virtual Screening (SBVS), allowed us to efficiently screen a wide database of natural compounds, thus identifying potential candidates with dual inhibitory activity against HDAC8 and tubulin. After compounds’ prioritizations based on their binding affinities and predicted selectivity profiles, we sought to find novel dual HDAC8/tubulin binders acting as potential therapeutic agents with improved efficacy and reduced off-target effects [68]. In particular, we focused our attention on natural compounds without chiral centers. This choice was adopted to overcome the problem linked to the incomplete stereochemical information in structure libraries that could lead to a “coin toss” regarding the presence of the “ideal” chiral structure [69].

Our virtual screening process yielded a subset of compounds with greater potential for further investigation. Specifically, CNP0112925 (arundinin) and CNP0217284 showed the greatest selectivity towards isoform 8 of HDAC, though only arundinin was confirmed to inhibit HDAC8 enzymatic activity and thus underwent functional investigation.

Arundinin has a polyphenolic structure, and it has already evaluated for its anti-inflammatory activities [54]. Indeed, it was able to induce the inhibition of superoxide anion generation and elastase release with an IC_50_ of about 0.9 µM.

Similarly to what was reported for recently synthesized dual HDAC/tubulin inhibitors capable of exerting anti-tumor activity via mitochondrial dysfunction and apoptosis induction [70], arundinin was able to impair in vitro breast cancer cell growth, promoting the accumulation of mitochondrial ROS and apoptosis, which was confirmed by the activation of Caspase 3 and PARP, the upregulation of BAX, a relevant effector of mitochondrial apoptosis, and the downregulation of anti-apoptotic BCL2 protein.

Taken together, these results suggest that arundinin represents a new promising hit as a dual HDAC8/tubulin inhibitor deserving further investigations in advanced preclinical models of human cancer.

Finally, this polyphenolic compound is particularly intriguing, as it is found in various Asian plants, including *Pleione yunnanensis* and *Arundina graminifolia*, both commonly used in traditional medicine. *A. graminifolia*, an evergreen terrestrial orchid, is believed to possess anti-arthritic, detoxifying, and anti-irritant properties. Phytochemical investigations of *A. graminifolia* have revealed the presence of sterols, stilbenoids, phenolic acids, and triterpenes, with stilbenoids emerging as the most significant secondary metabolites. These compounds are known for their antiviral, anti-tumor, antioxidant, and cytotoxic activities [71]. Another traditional remedy, *Pseudobulbus Cremastrae seu Pleiones* (PCsP), commonly known as “Shancigu” in China, is an herbal medicine that includes species such as *Pleione bulbocodioides*, *Cremastra appendiculata*, and *Pleione yunnanensis*. These species treat cancer, bacterial infections, and conditions like furuncles, carbuncles, scrofula, snake bites, and abdominal lumps [72,73]. Thus, understanding the pathways of bioactive compounds in these plants is crucial for developing improved semi-synthetic derivatives with better pharmacokinetic and pharmacodynamic properties, also expanding knowledge of these beneficial medicinal herbs.

## 5. Conclusions

In conclusion, this study highlights the potential of dual-target strategies, particularly leveraging natural compounds, in advancing cancer therapies. Our investigation has identified arundinin, a polyphenolic compound from traditional Asian medicinal plants, as a potent dual HDAC8/tubulin inhibitor with promising anti-cancer activity. Through computational screening and functional assays, arundinin exhibited high effectiveness in promoting apoptosis in breast cancer cells by inducing mitochondrial dysfunction and oxidative stress. The traditional medicinal use of arundin-containing plants and their diverse bioactive profiles further emphasize the relevance of natural compounds in drug discovery, particularly for diseases where traditional remedies have shown therapeutic promise. Further studies into arundinin’s efficacy in preclinical cancer models and its pharmacokinetic properties are essential to fully assess its potential as a novel anti-cancer treatment. Overall, this research strengthens the relevance of natural dual-target compounds and emphasizes traditional medicinal sources as foundations for innovation in oncology.

## Figures and Tables

**Table 1:** Best D-Score values, reported in kcal/mol, of the final *hits* for each HDAC isoform and tubulin acquired from the docking process. The cut-off is respected only on HDAC8 and tubulin.

Compound	D-Score (kcal/mol)				
	HDAC1	HDAC2	HDAC3	HDAC8	Tubulin
CNP0112925	−5.47	−7.64	−4.82	−8.11	−10.65
CNP0228436	−3.15	−5.01	−4.05	−8.05	−10.64
CNP0217284	−3.81	−7.65	−4.97	−8.34	−10.64
CNP0371079	−4.65	−8.45	−4.10	−8.48	−10.79

**Table 2:** Effects of compounds CNP0112925 (arundinin), CNP0217284, and Trichostatin A on HDAC8 activity.

Compound	HDAC8IC_50_ (μM) ^a^
CNP0112925	36.87 ± 4.29
CNP0217284	>300
Trichostatin A	0.35 ± 0.04

^a^ IC_50_ values are defined as the drug concentration that reduces target activity by 50% and is reported as a mean value of three determinations carried out in duplicate ± SEM.

## Data Availability

Data are contained in the article and Supplementary Materials.

## Supplementary Materials

The following supporting information can be downloaded at https://www.mdpi.com/article/10.3390/antiox13111427/s1: Table S1: RMSD values of redocking analysis on the two generated grids for HDAC2 and HDAC8. Table S2: RMSD and D-Score values of redocking analysis on the two generated grids for tubulin chains A/B and chains C/D. Table S3: Best docking score (D-Score) values of Trichostatin A and Vorinostat for each HDAC isoform. For all isoforms, the best value was obtained for Trichostatin A, and it was used as a cut-off for the subsequent SBVS. Table S4: 48 h IC_50_ values of Arundinin for MCF7 and MDA-MB-453 BC cell lines. Figure S1: MDs analysis for Vorinostat in complex with Class I HDACs. In the top panel, the ligand RMSD plot, expressed in Å and calculated on the heavy atoms of the ligand, is reported. In the bottom panel, ligand atom interactions with the protein residues of HDAC8 (light blue), HDAC1 (pink), HDAC2 (purple), and HDAC3 (orange) are indicated. Only interactions that occur more than 30.0% of the simulation time in 200 ns of the trajectory are shown. Figure S2: MDs analysis for Trichostatin A in complex with Class I HDACs. In the top panel, the ligand RMSD plot, expressed in Å and calculated on the heavy atoms of the ligand, is reported. In the bottom panel, ligand atom interactions with the protein residues of HDAC8 (light blue), HDAC1 (pink), HDAC2 (purple), and HDAC3 (orange) are indicated. Only interactions that occur more than 30.0% of the simulation time in 200 ns of the trajectory are shown. Figure S3: MDs analysis for CNP0228436 in complex with Class I HDACs. In the top panel, the ligand RMSD plot, expressed in Å and calculated on the heavy atoms of the ligand, is reported. In the bottom panel, ligand atom interactions with the protein residues of HDAC8 (light blue), HDAC1 (pink), HDAC2 (purple), and HDAC3 (orange) are indicated. Only interactions that occur more than 30.0% of the simulation time in 200 ns of the trajectory are shown. Figure S4: MDs analysis for CNP0371079 in complex with Class I HDACs. In the top panel, the ligand RMSD plot, expressed in Å and calculated on the heavy atoms of the ligand, is reported. In the bottom panel, ligand atom interactions with the protein residues of HDAC8 (light blue), HDAC1 (pink), HDAC2 (purple), and HDAC3 (orange)are indicated. Only interactions that occur more than 30.0% of the simulation time in 200 ns of the trajectory are shown. Figure S5: MDs analysis for arundinin (CNP0112925) in complex with HDAC8, performed in triplicate. In the top panel, the ligand RMSD plot, expressed in Å and calculated on the heavy atoms of the ligand, is reported. In the bottom panel, ligand atom interactions with the protein residues of HDAC8 are indicated. Only interactions that occur more than 30.0% of the simulation time in 200 ns of the trajectory are shown. Figure S6: MDs analysis for arundinin (CNP0112925) in complex with tubulin, performed in triplicate. In the top panel, the ligand RMSD plot, expressed in Å and calculated on the heavy atoms of the ligand, is reported. In the bottom panel, ligand atom interactions with the protein residues of tubulin are indicated. Only interactions that occur more than 30.0% of the simulation time in 200 ns of the trajectory are shown.
