# Supplementary material for: Hit Identification and Functional Validation of Novel Dual Inhibitors of HDAC8 and Tubulin Identified by Combining Docking and Molecular Dynamics Simulations
Source: Antioxidants (Basel). 2024 Nov 20;13(11):1427. doi: 10.3390/antiox13111427 (PMC11591096; doi:10.3390/antiox13111427)
Supplement: Supplementary file 1 [file antioxidants-13-01427-s001.zip › antioxidants-3306530-supplementary.pdf]

Supplementary Material for

Hit Identification and functional validation of novel dual inhibitors of HDAC8 and tubulin identified by combining docking and Molecular Dynamics simulations

HDAC isoform	RMSD (Å)	
	**Co-crystallized ligand grid**	**His-His-Tyr grid**
HDAC2 (Vorinostat)	1.21	1.18
HDAC8 (Trichostatin A)	2.25	0.40

Table S1: RMSD values of redocking analysis on the two generated grids for HDAC2 and HDAC8.

α/β dimer	RMSD (Å)	D-Score (kcal/mol)
chains A/B	0.79	-10.55
chains C/D	4.54	-9.54

Table S2: RMSD and D-Score values of redocking analysis of colchicine on the two generated grids for tubulin chains A/B and chains C/D.

HDAC isoform	D-Score (kcal/mol)	
	**Vorinostat**	**Trichostatin A**
HDAC1	-3.05	-5.56
HDAC2	-6.87	-8.69
HDAC3	-2.86	-6.71
HDAC8	-4.56	-7.92

Table S3: Best D-Score values of Trichostatin A and Vorinostat for each HDAC isoform, acquired from docking process. For all isoforms the best value was obtained for Trichostatin A and it was used as a *cut-off* for the subsequent SBVS.

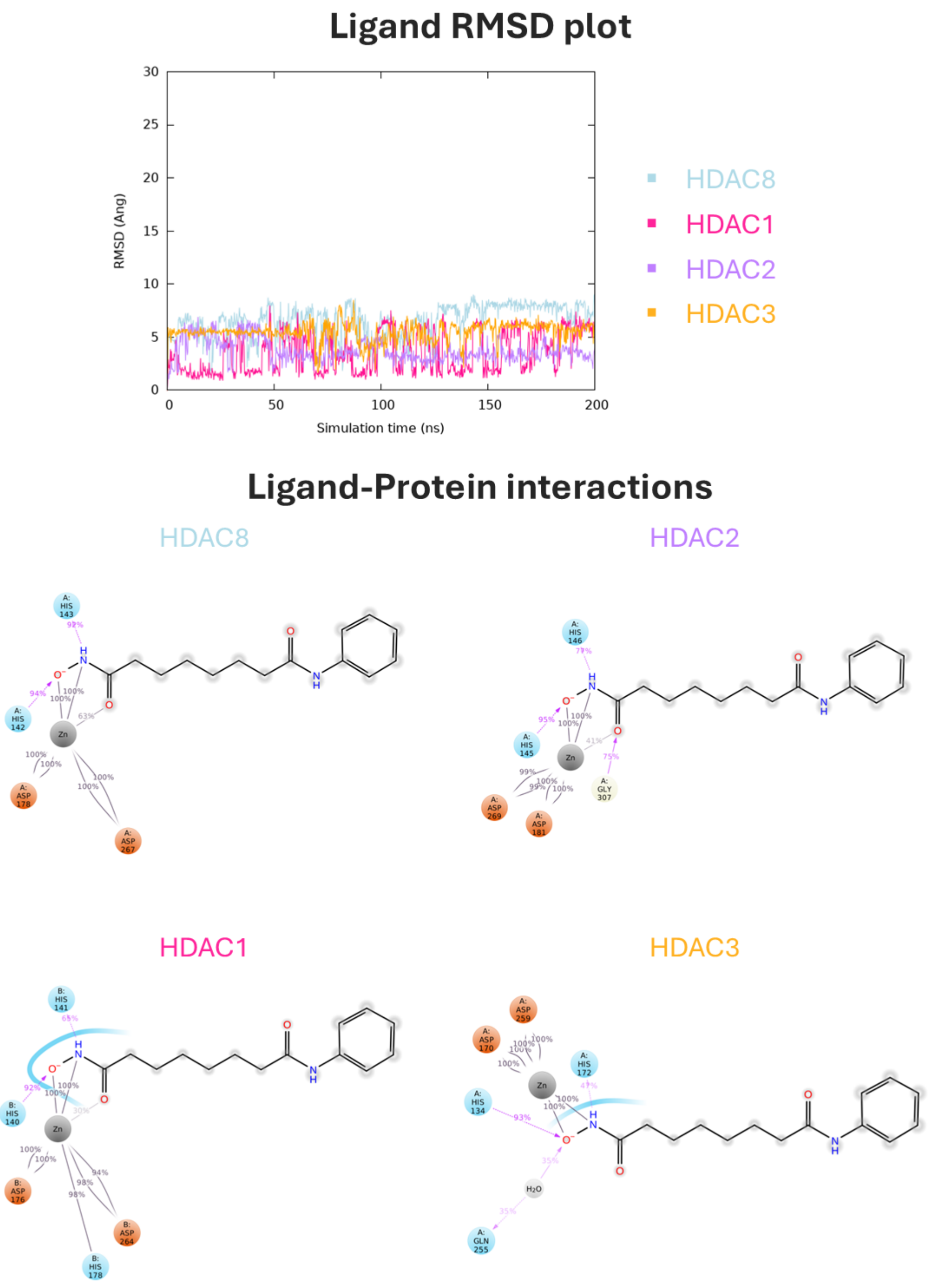

Figure S1: MDs analysis for Vorinostat in complex with Class I HDACs. In the top panel, the ligand RMSD plot, expressed in Å and calculated on the heavy atoms of the ligand, is reported. In the bottom panel, ligand atom interactions with the protein residues of HDAC8 (light-blue), HDAC1 (pink), HDAC2 (purple) and HDAC3 (orange) are indicated. Only interactions that occur more than 30.0% of the simulation time in 200 ns of the trajectory are shown.

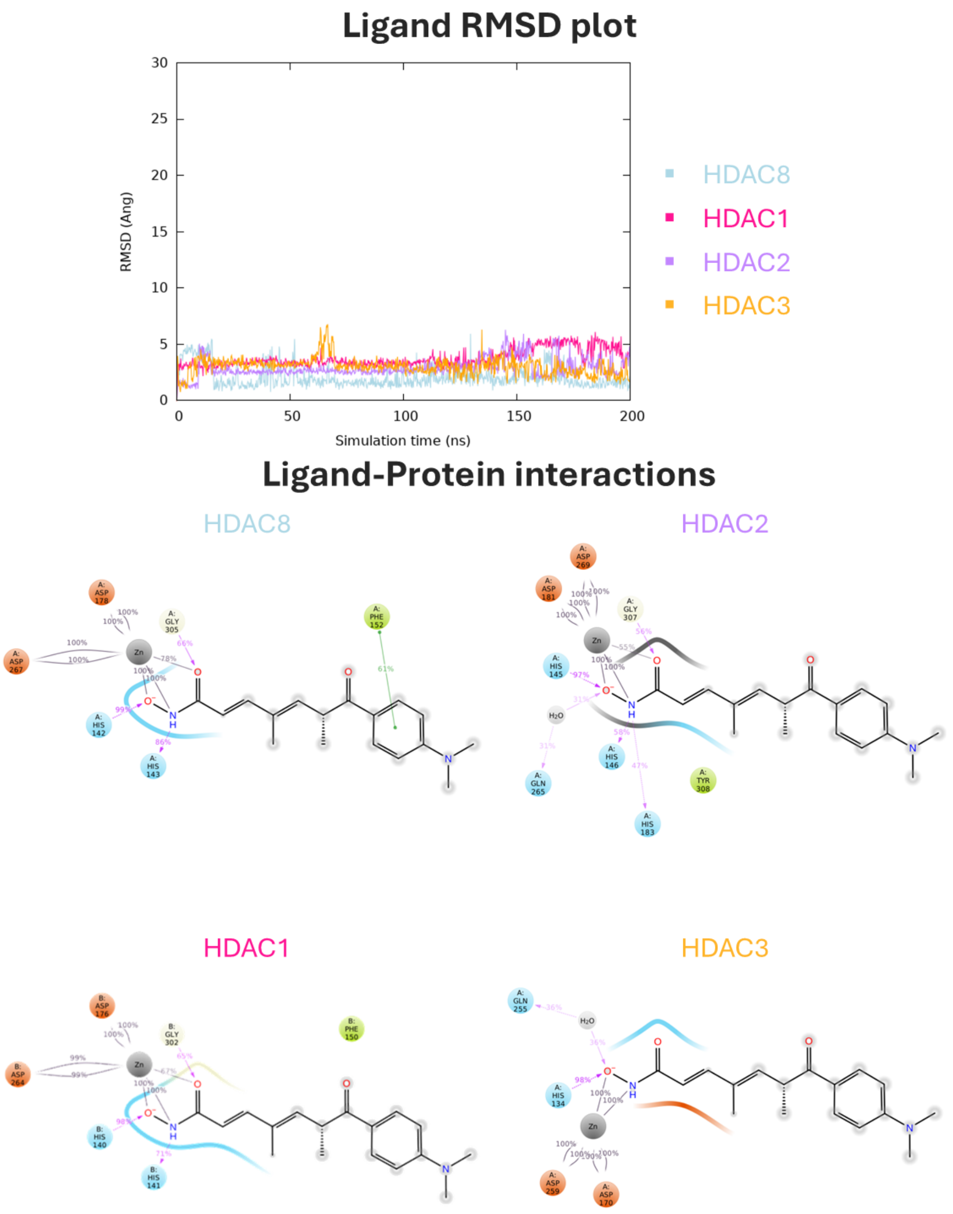

Figure S2: MDs analysis for Trichostatin A in complex with Class I HDACs. In the top panel, the ligand RMSD plot, expressed in Å and calculated on the heavy atoms of the ligand, is reported. In the bottom panel, ligand atom interactions with the protein residues of HDAC8 (light-blue), HDAC1 (pink), HDAC2 (purple) and HDAC3 (orange) are indicated. Only interactions that occur more than 30.0% of the simulation time in 200 ns of the trajectory are shown.

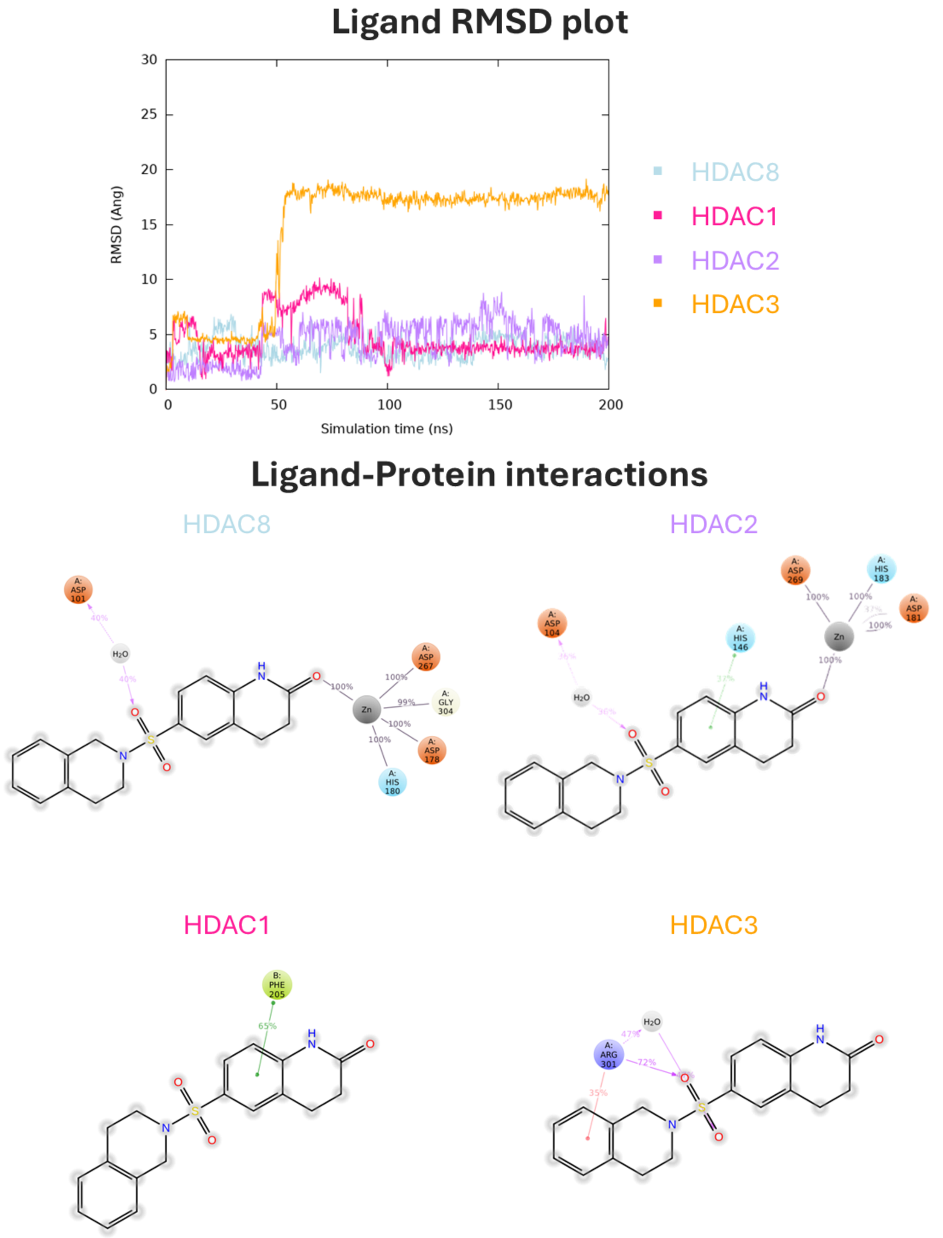

Figure S3: MDs analysis for CNP0228436 in complex with Class I HDACs. In the top panel, the ligand RMSD plot, expressed in Å and calculated on the heavy atoms of the ligand, is reported. In the bottom panel, ligand atom interactions with the protein residues of HDAC8 (light-blue), HDAC1 (pink), HDAC2 (purple) and HDAC3 (orange) are indicated. Only interactions that occur more than 30.0% of the simulation time in 200 ns of the trajectory are shown.

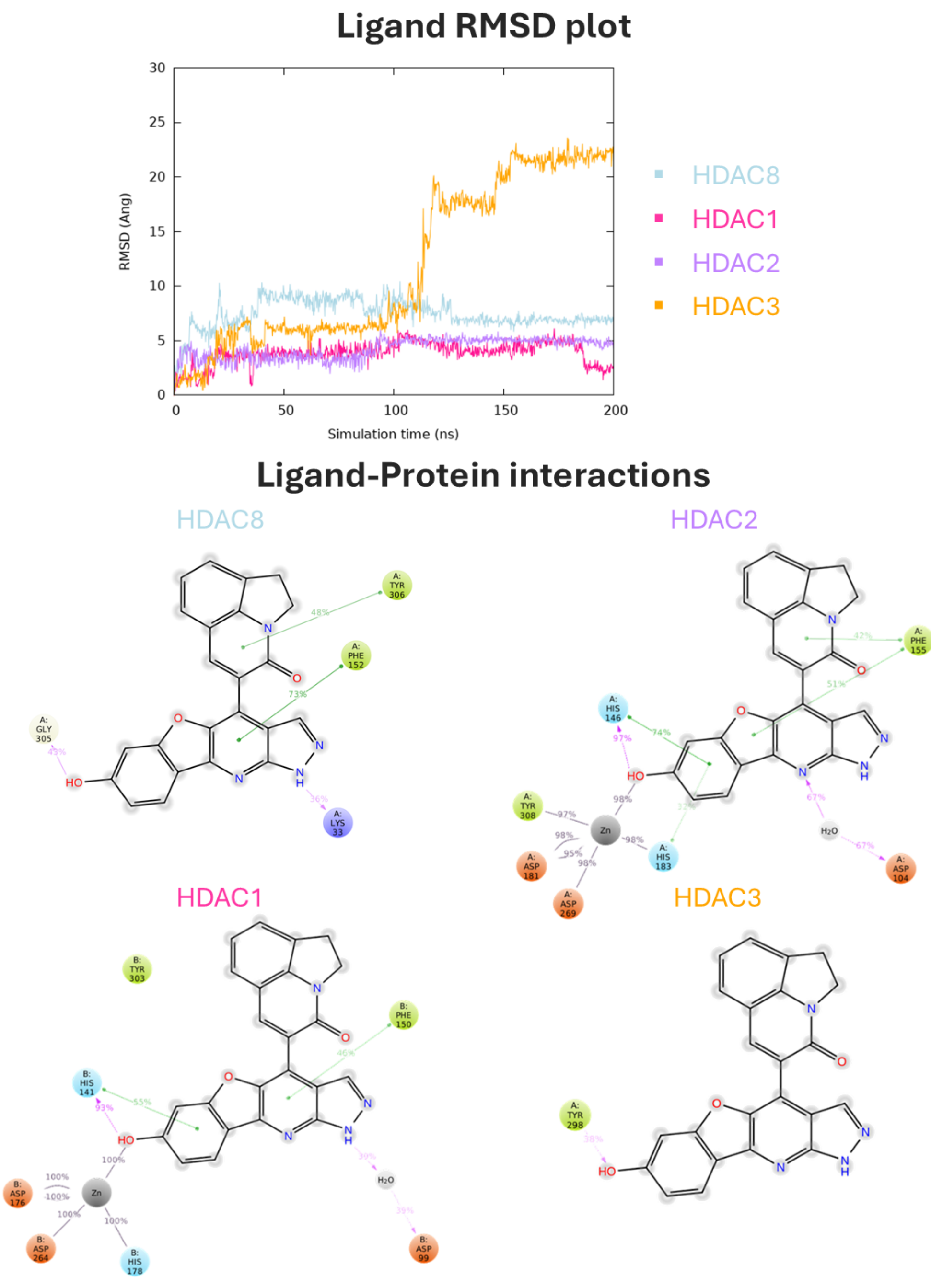

Figure S4: MDs analysis for CNP0371079 in complex with Class I HDACs. In the top panel, the ligand RMSD plot, expressed in Å and calculated on the heavy atoms of the ligand, is reported. In the bottom panel, ligand atom interactions with the protein residues of HDAC8 (light-blue), HDAC1 (pink), HDAC2 (purple) and HDAC3 (orange) are indicated. Only interactions that occur more than 30.0% of the simulation time in 200 ns of the trajectory are shown.

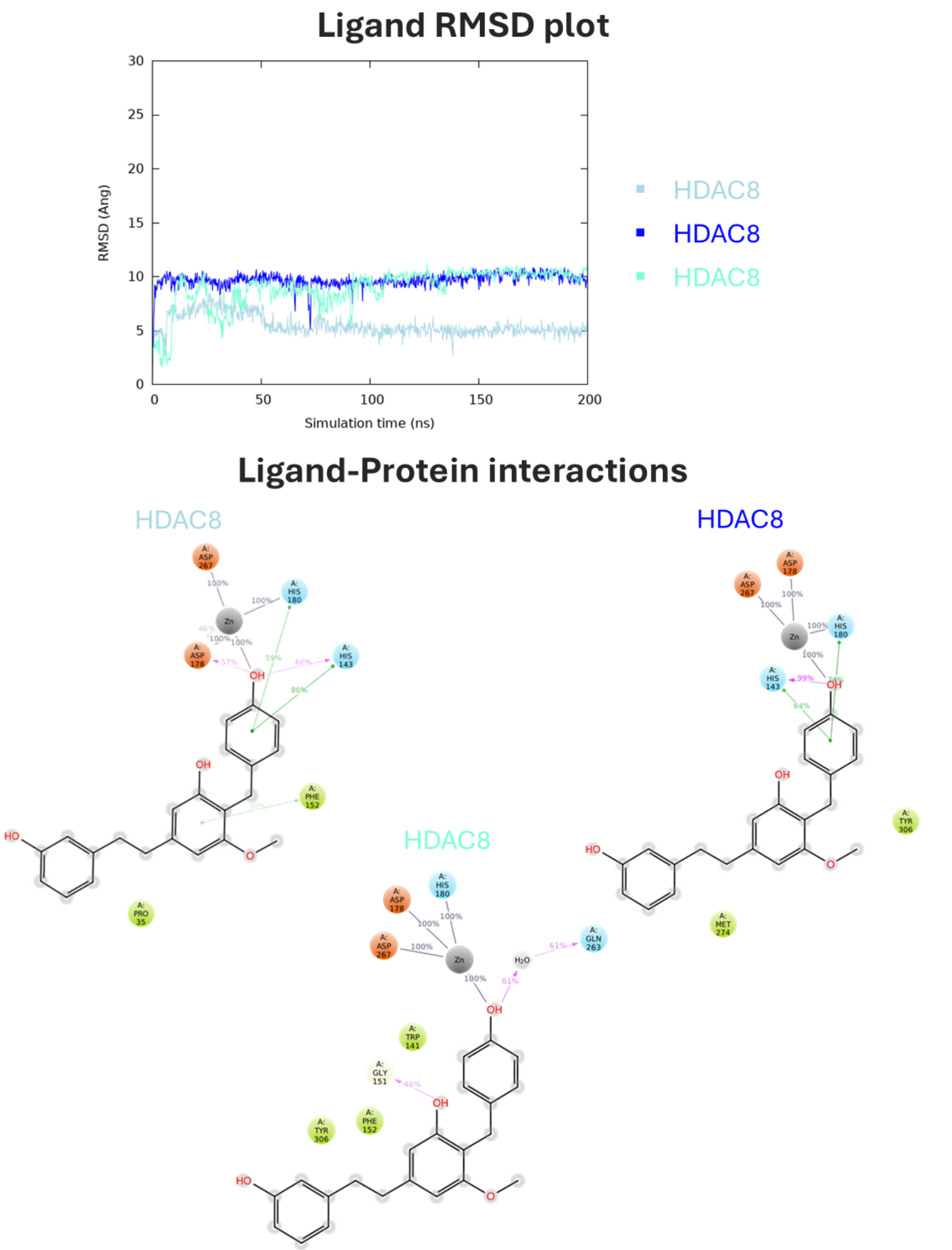

Figure S5: MDs analysis for Arundinin (CNP0112925) in complex with HDAC8, performed in triplicate. In the top panel, the ligand RMSD plot, expressed in Å and calculated on the heavy atoms of the ligand, is reported. In the bottom panel, ligand atom interactions with the protein residues of HDAC8 are indicated. Only interactions that occur more than 30.0% of the simulation time in 200 ns of the trajectory are shown.

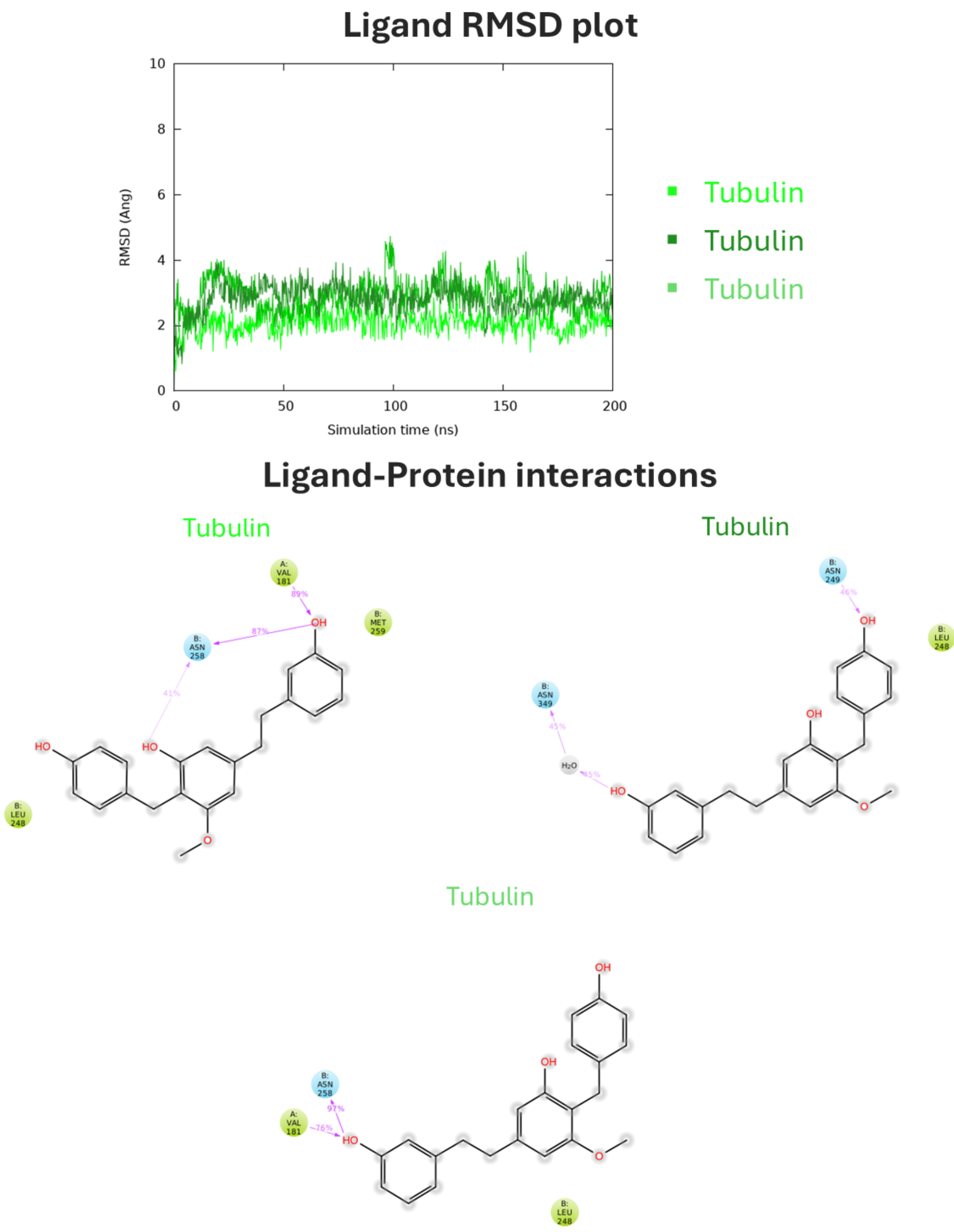

Figure S6: MDs analysis for Arundinin (CNP0112925) in complex with tubulin, performed in triplicate. In the top panel, the ligand RMSD plot, expressed in Å and calculated on the heavy atoms of the ligand, is reported. In the bottom panel, ligand atom interactions with the protein residues of tubulin are indicated. Only interactions that occur more than 30.0% of the simulation time in 200 ns of the trajectory are shown.

	IC_{50} (µM)
**MCF7**	**40.8**
**MDA-MB-453**	**71.74**

Table S4: 48h IC_{50} values of Arundinin for MCF7 and MDA-MB-453 BC cell lines.
